# Multistate Outbreak of *Salmonella* Thompson Infections Linked to Seafood Exposure — United States, 2021

**DOI:** 10.15585/mmwr.mm7219a2

**Published:** 2023-05-12

**Authors:** Ann Q. Shen, Alyssa Dalen, Laura Bankers, Shannon R. Matzinger, Colin Schwensohn, Kane Patel, Kelley B. Hise, Evelyn Pereira, Jennifer Cripe, Rachel H. Jervis

**Affiliations:** ^1^Colorado Department of Public Health and Environment; ^2^Division of Foodborne, Waterborne, and Environmental Diseases, National Center for Emerging and Zoonotic Infectious Diseases, CDC; ^3^Oak Ridge Institute for Science and Education, Oak Ridge, Tennessee; ^4^Food and Drug Administration, Silver Spring, Maryland.

In July 2021, the Colorado Department of Public Health and Environment (CDPHE) laboratory identified a cluster of five *Salmonella enterica* serotype Thompson isolates related to one another within one allele difference, using whole genome multilocus sequence typing (wgMLST). These five isolates, submitted to the public health laboratory as is routine process for confirmatory testing of *Salmonella*, were highly related to those identified in a 2020 multistate investigation, during which traceback was conducted for sushi-grade tuna and salmon; a common supplier was not identified. The 2021 investigation commenced on August 5, 2021, with five patients living in Colorado, and one each in Missouri, Washington, and Wisconsin. During August–December 2021, CDC, CDPHE, public health and regulatory officials in several states, and the Food and Drug Administration (FDA) conducted epidemiologic, environmental, and laboratory investigations of this multistate outbreak of *Salmonella* Thompson. Isolates were genetically related to one another and to 2020 isolates within zero to one allele difference. Implicated seafood products were traced to a single seafood distributor, in which the outbreak strain was identified through environmental sampling, and in which inspection identified inadequate sanitization and opportunities for cross-contamination of raw fish. The distributor issued a voluntary recall of 16 seafood items with high potential for contamination and completed remediation actions. This outbreak illustrated the importance of effective cleaning and sanitizing procedures and implementation of controls. When multiple products are recalled during an outbreak investigation, collaboration between public health agencies and implicated facilities can help provide food safety information to restaurants, retailers, and consumers, and to ensure disposal of all recalled products.

## Epidemiologic Investigation

A case was defined as an infection with a *S.* Thompson isolate within three allele differences of the outbreak strain by wgMLST, and with illness onset during May 11–October 16, 2021. Local or state health departments first contacted patients to attempt a routine interview to collect information on exposures during the week preceding illness onset, symptoms, and outcomes; seafood consumption was commonly reported. Consequently, a seafood-focused supplementary questionnaire was deployed on August 13, 2021, to reinterview patients. This activity was reviewed by CDC and was conducted consistent with applicable federal law and CDC policy.*

**FIGURE 1:**
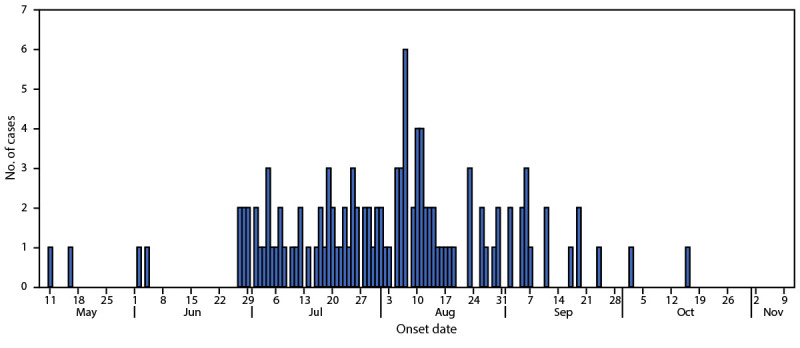
Illness onset dates* of laboratory-confirmed, outbreak-associated cases of *Salmonella* Thompson (N = 115) — 15 U.S. states,^†^ May–October, 2021^§^ * Estimated when clinical data were not available. ^†^ Arizona, California, Colorado, Connecticut, Iowa, Minnesota, Missouri, Nebraska, New Jersey, Pennsylvania, Texas, Virginia, Washington, Wisconsin, and Wyoming. ^§^ As of December 2, 2021.

**FIGURE 2:**
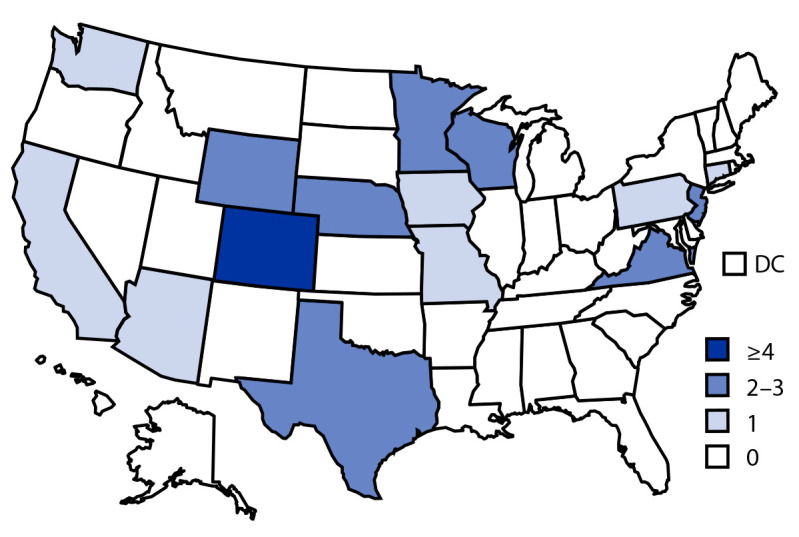
Number of persons infected with the outbreak strain of *Salmonella* Thompson, by state of residence and number of cases per state (N = 115) — United States, May–October 2021 **Abbreviation: **DC = District of Columbia.

A total of 115 outbreak-related cases were reported from 15 states (Figure 1). Among these, 93 (81%) patients were Colorado residents and 22 (19%) lived in 14 additional states (Figure 2). All but eight patients reported travel to Colorado during their exposure period (the 7 days before illness onset). All 2021 calendar-year isolates were within zero to two allele differences by wgMLST, and were zero to one allele difference from the 2020 S. Thompson outbreak isolates. The median age of patients in the 2021 outbreak was 29 years (range = <1–85 years); 61 (53%) were female. Among 111 patients with information available, illness onset occurred during May 11–October 16. Twenty (26%) patients were hospitalized; no deaths were reported. Among 90 patients with exposure information, 76 (84%) reported eating any seafood, and 35 of 85 (41%) reported consuming sushi or raw fish. Both proportions were significantly higher than the proportion of respondents from the FoodNet Population Survey that reported eating any seafood (35%) or sushi or raw fish (8%) during the week before participating in the survey (*1*). FoodNet Population Survey data provide estimates on how often persons in the surveillance area are typically expected to be exposed to a specific food vehicle; deviations from the expected estimate might indicate that a food vehicle should be considered suspect.

## Environmental Health Investigation

CDPHE and FDA completed concurrent traceback and traceforward investigations. Traceback investigation involved requesting invoices from all restaurants where patients reported having dined during their exposure period. Records related to tuna, shrimp, salmon, and halibut were reviewed to ascertain common distribution sources. In addition, investigators identified six subclusters, defined as establishments where two or more patients from separate households reported consuming a variety of seafood items during their exposure period. Detailed build sheets (comprehensive instructional documents for food preparation, storage, and plating) from the six subclusters were collected. Five subcluster establishments were restaurants, including one sushi bar located within a grocery store. Traceback identified a common seafood distributor and processor that directly supplied four of the six subcluster establishments.

CDPHE’s Division of Environmental Health Services (DEHS) conducted a traceforward investigation. DEHS collected customer lists and five samples of three unique products from the implicated seafood distributor and processor facility. The outbreak strain was not identified in these five samples.

On September 22, 2021, FDA performed a seafood Hazard Analysis Critical Control Point inspection^†^ with environmental sampling at the implicated distributor and processor facility. The outbreak strain was identified in 13 (9.8%) of 132 environmental swabs of the facility’s floor and floor drains. The inspection also identified several opportunities for cross-contamination of raw fish, including the use of high pressure hoses that produced backsplash onto fresh product. Other substantial findings included insufficient sanitizer concentration, condensation dripping onto product contact surfaces, and using gloved hands to remove water from floor drains without changing gloves after contact with the drains (*2*).

## Laboratory Investigation

Whole genome sequencing (WGS) was performed on the 21 Colorado isolates from 2020 and 97 of the 2021 isolates at the CDPHE laboratory following the PulseNet or Illumina MiSeq paired-end WGS standard operating procedure (*3*). Assembly and analysis of these isolates was performed using BioNumerics, a software application for managing microbiologic data, following PulseNet guidelines.^§^ Results were submitted to the PulseNet National Database, which was then queried to pull WGS data for all outbreak-associated samples and environmental samples collected by FDA (*4*). wgMLST was performed on each genome using BioNumerics to generate a similarity matrix (a matrix of the number of allele differences for all possible pairwise comparisons). A clustering analysis was conducted on the matrix using the unweighted pair group method with arithmetic mean algorithm, a method for constructing dendrograms in a stepwise manner, based on order of similarity (Supplementary Figure, https://stacks.cdc.gov/view/cdc/127756).

Overall, WGS results supported the epidemiologic data, indicating a common source of infection for the 2020 and 2021 outbreaks, confirmed in 2021 to be a common seafood distributor and processor. Based on genetic similarity, the largest grouping of outbreak-associated isolates contained 100 samples, including clinical isolates from 2020 and 2021, and nine environmental isolates from 2021, all with zero allele difference. Among 112 clinical isolates, WGS analysis results indicated no concerning antibiotic resistance, signifying no impact on provider treatment recommendations.

## Public Health Response

On October 8, 2021, the seafood distributor and processor issued a voluntary recall of 16 raw, fresh seafood products processed onsite (*5*). Potential cross-contamination within the processing environment resulted in the large number of recalled products and compounded the challenge of identifying a specific food vehicle during the investigation. A call with the facility occurred on October 7 to discuss the WGS results, recall, and corrective actions. On October 8, the facility temporarily ceased operations to carry out corrective actions, which included reassessing cleaning and sanitizing procedures and intensifying cleaning and sanitizing of the facility and equipment, eliminating the use of high-pressure hoses, hiring a food safety consultant to assist with the facility’s root cause analysis, and revising the environmental monitoring program . On October 8, FDA (*6*), CDC (*7*), and CDPHE (*8*) all issued web posts alerting the public to this outbreak and identifying the facility. FDA carried out additional traceback actions using information collected from the facility regarding suppliers. Multiple upstream domestic and foreign seafood suppliers were identified, but evidence was not available to identify a single contamination source.

CDC, FDA, and CDPHE advised consumers, restaurants, and retailers not to eat, sell, or serve any recalled seafood that was sold in three Colorado grocery stores and several restaurants. Public messaging advised consumers to discard any recalled products they might have purchased fresh and subsequently frozen.

## Discussion

The outbreak investigation resulted in a large amount of interview, traceback, and traceforward data, compounding the challenge of identifying a specific food vehicle or source, and limiting the findings. For example, many patients reported eating seafood items at a variety of locations during their exposure periods. Subcluster analysis allowed investigators to focus this information and prioritize data collection from facilities commonly reported by patients. However, the small size and complex nature of each subcluster limited the power of this analysis. The largest subcluster contained only four confirmed outbreak patients, and three subclusters included only two patients each. In one instance, a single patient reported consuming food at more than one subcluster facility, which further complicated the analysis. Reinterviewing patients and asking about dining history at commonly reported restaurants might be beneficial to expanding the size of subclusters and power of data collected. Throughout the investigation, competing priorities limited the capacity of public health agencies, leading to delays in collection of invoices, build sheets, and customer lists. Traceforward actions allowed investigators to supplement supplier information collected by local public health and regulatory agencies with customer lists provided by distributors to identify common distribution patterns among reported facilities. In addition, investigators faced resistance from restaurant facilities concerned about sharing proprietary information on dishes or supplier invoices.

This investigation illustrates the importance of clear and direct communication to the public and partnerships with local public health and regulatory officials who can leverage existing relationships with local facilities. When an outbreak investigation results in the recall of multiple products, collaboration between public health agencies and implicated facilities can serve to provide food safety information to restaurants, retailers, and consumers, and to ensure disposal of all recalled products. Implementing appropriate cleaning practices at processing facilities that are focused on prevention of cross-contamination is important.

**Summary**

**What is already known about this topic?**

*Salmonella* Thompson is a relatively uncommon serotype not typically associated with seafood. Previous outbreaks have been associated with beef, chicken, and vine-stalk and leafy vegetables.

**What is added by this report?**

During May–October 2021, 115 persons in 15 states became ill with *S.* Thompson. Most patients reported seafood consumption in Colorado before illness onset. The outbreak strain was identified at a single seafood distributor and processor in Denver, where opportunities for cross-contamination, due to inadequate sanitization, were identified.

**What are the implications for public health practice?**

Cleaning practices at processing facilities must prevent cross-contamination. Multiagency collaboration to provide food safety information during product recalls is essential to ensure disposal of all recalled products.

## Footnotes

* 45 C.F.R. part 46.102(l)(2), 21 C.F.R. part 56; 42 U.S.C. Sect. 241(d); 5 U.S.C. Sect. 552a; 44 U.S.C. Sect. 3501 et seq.

† A management system in which food safety is addressed through the analysis and control of biologic, chemical, and physical hazards from raw material production, procurement and handling, to manufacturing, distribution, and consumption of the finished product. https://www.fda.gov/food/guidance-regulation-food-and-dietary-supplements/hazard-analysis-critical-control-point-haccp

§ PulseNet Standard Operating Procedure for the Organism-Specific Surveillance Database Workflow. Doc. No. PND05, Ver. No. 01, Effective date: June 23, 2020.
